# Electrical Pacing of Cardiac Tissue Including Potassium Inward Rectification

**DOI:** 10.1371/journal.pone.0127837

**Published:** 2015-06-09

**Authors:** Suran Galappaththige, Bradley J. Roth

**Affiliations:** Department of Physics, Oakland University, Rochester, Michigan, United States of America; Gent University, BELGIUM

## Abstract

In this study cardiac tissue is stimulated electrically through a small unipolar electrode. Numerical simulations predict that around an electrode are adjacent regions of depolarization and hyperpolarization. Experiments have shown that during pacing of resting cardiac tissue the hyperpolarization is often inhibited. Our goal is to determine if the inward rectifying potassium current (*I_K1_*) causes the inhibition of hyperpolarization. Numerical simulations were carried out using the bidomain model with potassium dynamics specified to be inward rectifying. In the simulations, adjacent regions of depolarization and hyperpolarization were observed surrounding the electrode. For cathodal currents the virtual anode produces a hyperpolarization that decreases over time. For long duration pulses the current-voltage curve is non-linear, with very small hyperpolarization compared to depolarization. For short pulses, the hyperpolarization is more prominent. Without the inward potassium rectification, the current voltage curve is linear and the hyperpolarization is evident for both long and short pulses. In conclusion, the inward rectification of the potassium current explains the inhibition of hyperpolarization for long duration stimulus pulses, but not for short duration pulses.

## Introduction

Cardiovascular disease is the leading cause of death in the United States. Many cardiac arrhythmias are treated by electrical stimulation, using either a pacemaker or a defibrillator. In order to optimize these treatments, we must understand how an electrical stimulus interacts with cardiac tissue. One of the simplest ways to stimulate cardiac tissue is to pass current through a single, small, extracellular electrode, known as unipolar stimulation [1]. Theoretical simulations using the passive anisotropic bidomain model have been used to study the electrical behavior of the cardiac muscle when stimulated by a unipolar electrode [2]. These simulations predict that the transmembrane potential (*V*_*m*_) throughout a two dimensional sheet of cardiac tissue in response to cathodal stimulation creates a region of depolarization under the cathode that has a “dog-bone” shape oriented perpendicular to the fiber direction, and regions of hyperpolarization parallel to the fiber direction. Anodal stimulation results in regions of hyperpolarization under the electrode and adjacent regions of depolarization.

Experiments in which a stimulus is applied to refractory tissue produce results similar to the predictions of the passive bidomain model, with adjacent regions of depolarization and hyperpolarization [3–5]. However, when a stimulus is applied to resting (diastolic) tissue, hyperpolarization is rarely seen [6–8]. One reason for this behavior might be that the shock excited an action potential resulting in active depolarization that masks any hyperpolarization. Another reason, however, might be the inward rectification of the resting membrane caused by the *I*_*K1*_ potassium current [9].

The *I*_*K1*_ current allows potassium to selectively cross the cell membrane. Normally a potassium current is outwardly rectifying because there is more potassium inside a cell than outside, so the outward current is larger than the inward current. However, *I*_*K1*_ is known as an anomalous rectifier, because it behaves in the opposite way: the inward current is larger than the outward current. *I*_*K1*_ is one of the dominant currents when the transmembrane potential is near or below its resting potential.

Sambelashvili et al. [10] studied subthreshold stimulation of diastolic cardiac tissue using a unipolar electrode. They observed less hyperpolarization than depolarization in their experiments, and found that they could explain the difference between the response to cathodal and anodal stimuli using the Luo-Rudy model of ion channel kinetics [11]. They concluded that the *I*_*K1*_ current was the main cause of this polarity dependence. However, their calculations included other membrane currents—such as sodium and calcium currents as well as other potassium currents—that may also contribute to the behavior.

In this study, our goal is to determine if the *I*_*K1*_ current alone, without any contribution whatsoever from other active currents, can explain how the resulting depolarization and hyperpolarization depend on stimulus polarity, strength, and duration. In addition, we analyze how the transmembrane potential evolves in time.

## Methods

The cardiac tissue is modeled as a two-dimensional bidomain [12]. The anisotropy of the tissue is characterized by four parameters, the intracellular and extracellular conductivities in the directions parallel and perpendicular to the fiber orientation. The conductivity values are the same as used by Sepulveda et al. [2] so that the tissue has unequal anisotropy ratios. The bidomain model is given as a pair of coupled partial differential equations

$$g_{ex}\;\frac{\partial^{2}V_{e}}{\partial x^{2}}+g_{ey}\;\frac{\partial^{2}V_{e}}{\partial y^{2}}=-\beta [C_{m}\frac{\partial V_{m}}{\partial t}+G_{m}{(V}_{m}-E_{K1})+I_{K1}]+I_{stm}$$ (1)

$$(g_{ix}+g_{ex})\frac{\partial^{2}V_{e}}{\partial x^{2}}+(g_{iy}+g_{ey})\frac{\partial^{2}V_{e}}{\partial y^{2}}+g_{ix}\;\frac{\partial^{2}V_{m}}{\partial x^{2}}+g_{iy}\;\frac{\partial^{2}V_{m}}{\partial y^{2}}=I_{stm}$$ (2)

where *V*_*m*_ and *V*_*e*_ are the transmembrane and extracellular potentials. Table 1 gives the tissue properties. The Nernst potential for potassium, *E*_*K1*_, is obtained from Eq 5. *C*_*m*_ is the membrane capacitance per unit area (0.01 μF/mm^2^), *G*_*m*_ is the linear membrane conductance (0.001 mS/mm^2^), and *β* is the surface-to-volume ratio (200 mm^-1^). For a current source of magnitude *I*_0_ (mA/mm) applied from a point electrode in the extracellular domain at the center of the tissue, *I*_*stm*_ is

$$I_{stm}=I_{0}\delta \left(x\right)\delta \left(y\right).$$ (3)

A positive *I*_*stim*_ is a cathode, and a negative *I*_*stim*_ is an anode.

**Table 1:** Tissue properties.

	Conductivity (mS/mm)
*g*_*ix*_	0.2
*g*_*iy*_	0.02
*g*_*ex*_	0.8
*g*_*ey*_	0.2

The time independent potassium current (*I*_*K1*_) was described by Luo and Rudy [11]

$$I_{K1}=G_{K1}\;K1_{\infty }\;\left(V_{m}-E_{K1}\right),$$ (4)

where

$$E_{K1}=\left(RT/F\right)\;\ln\left({\left[K^{+}\right]}_{o}/{\left[K^{+}\right]}_{i}\right).$$ (5)

Standard potassium concentrations are [*K* ^+^]_*o*_ (5.4 mmol/L) in the extracellular space and [*K* ^+^]_*i*_ (145 mmol/L) in the intracellular space. The molar gas constant is *R* (8.314 J/K mol), Faraday’s constant is *F* (96485 C/mol) and the absolute temperature is *T* (308 K) resulting in *E*_*K1*_ = -87 mV. The maximum conductance is *G*_*K*1_,

$$G_{K1}=0.0075\sqrt{{\left[K^{+}\right]}_{o}/5.4}\;\text{mS}/{\text{mm}}^{2},$$ (6)

the steady-state value of inactivation gate of the *I*_*K1*_ channel (*K*1_∞_) is

$$K1_{\infty }=\frac{\alpha_{K1}}{\beta_{K1}+\alpha_{K1}},$$ (7)

and the rate constants *α*_*K*1_ and *β*_*K*1_ (in 1/ms) are,

$$\alpha_{K1}=\frac{1.02}{1+e^{\left[0.2385\left(V_{m}-E_{K1}-59.215\right)\right]}}$$ (8)

$$\beta_{K1}=\frac{0.49124\;e^{\left[0.08032\left(V_{m}-E_{K1}+5.476\right)\right]}+e^{\left[0.06175\left(V_{m}-E_{K1}+594.31\right)\right]}}{1+e^{\left[-0.5143\left(V_{m}-E_{K1}+4.753\right)\right]}}.$$ (9)

**Fig 1:**
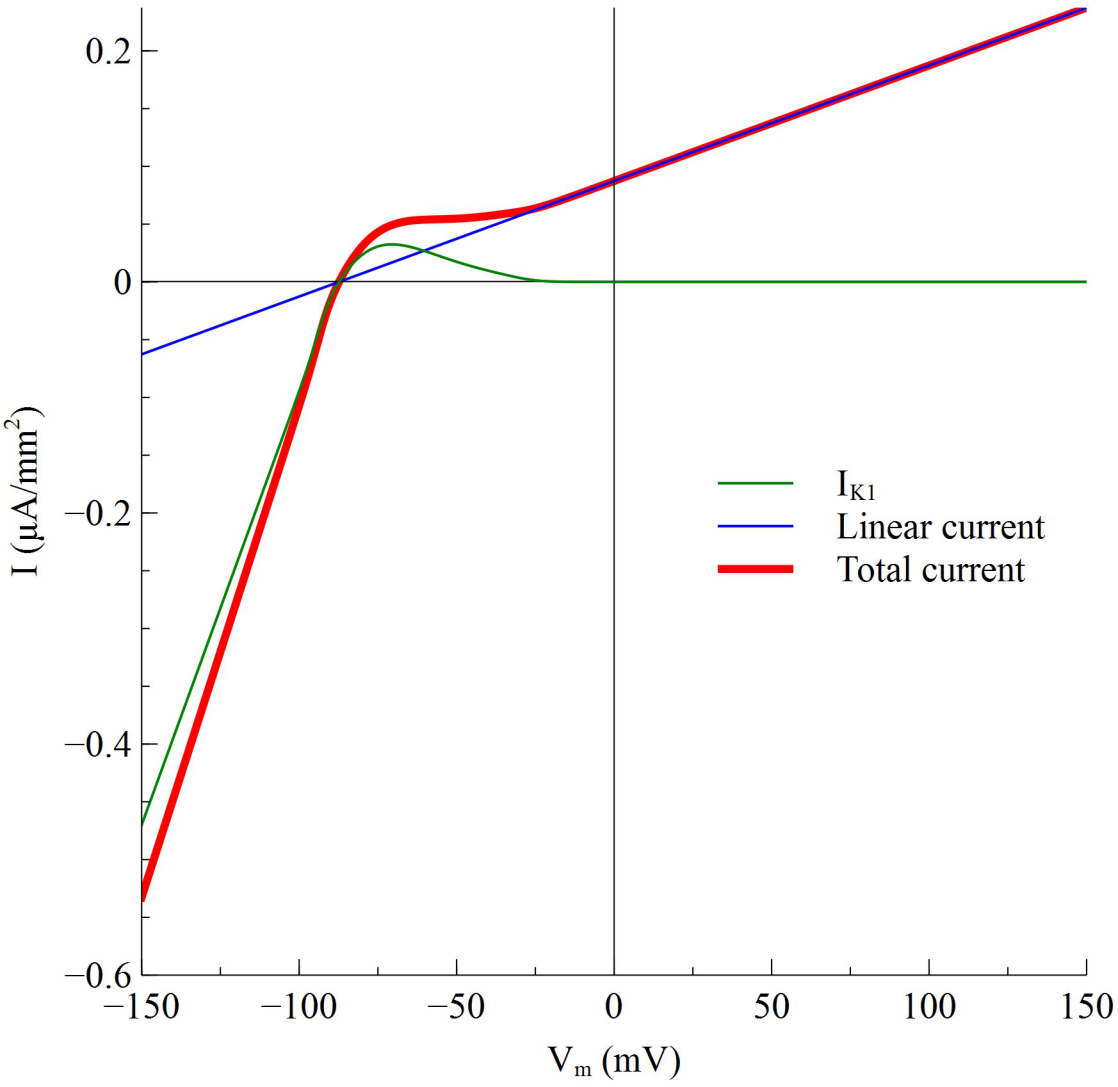
*I_K1_* (green), the linear current (blue), and the total membrane ionic current (red) versus *V_m_*.

In parallel with *I*_*K1*_ is a relatively small, passive membrane current *G*_*m*_ (*V*_*m—*_ *E*_*K1*_). Both currents are time-independent; they do not depend on the opening and closing of the ion channels. The variation of these currents with *V*_*m*_ is shown in Fig 1. The magnitude of the membrane current is inward and large for hyperpolarization (*V*_*m*_ < -87 mV), and outward and small for depolarization (*V*_*m*_ > -87 mV), as expected for a current showing inward rectification. At rest (*V*_*m*_ = -87 mV) the membrane current is zero.

We included the current *G_m_(V_m—_E_K1_)* for three reasons. First, adding this current is realistic because in the Luo-Rudy model [11] the total time-independent potassium current—the sum of *I_K1_*, a time-independent [K]_o_-insensitive current *I_Kp_*, and a background current *I_b_*—has this qualitative behavior. Second, when we performed the simulations without this current present, the membrane current was vanishingly small at large depolarizations (Fig 1), implying a very small membrane conductance and a large membrane time constant (*C_m_/G_m_*); the simulations took an unrealistically long time to approach steady state. This problem was eliminated by including the linear term in the membrane current. The membrane conductance was chosen small enough that it had little effect on the results for hyperpolarization and weak depolarization, but was large enough to result in a reasonable time constant for strong depolarizations (10 ms) [13]. Third, including a passive current makes comparing results with and without *I_K1_* easier; when *I_K1_* is not included *G_m_(V_m—_E_K1_)* is the only membrane current.

The partial differential equations describing the bidomain model are solved using numerical methods [14]. Starting with an initial value of *V*_*e*_(*t*), we solve Eq 1 for *V*_*m*_(*t+∆t*). Then we solve Eq 2 for *V*_*e*_(*t+∆t*), using the value of *V*_*m*_(*t+∆t*) in the source term, by the method of successive overrelaxation (SOR). In order to accelerate the convergence of the system, an overrelaxation parameter of *w =* 1.8 was used [15]. The iterative loop terminates when changes in *V*_*e*_ between subsequent iterations is less than 1 μV. All programs are written in Fortran 90 and compiled using the gfortran compiler (www.gcc.gnu.org) (the code is provided as S1 File). The space step is 0.1 mm both parallel and perpendicular to the fiber direction. The time step is 1 μs. The number of nodes in each direction is 100 and the tissue size is 10 mm × 10 mm.

## Results

**Fig 2:**
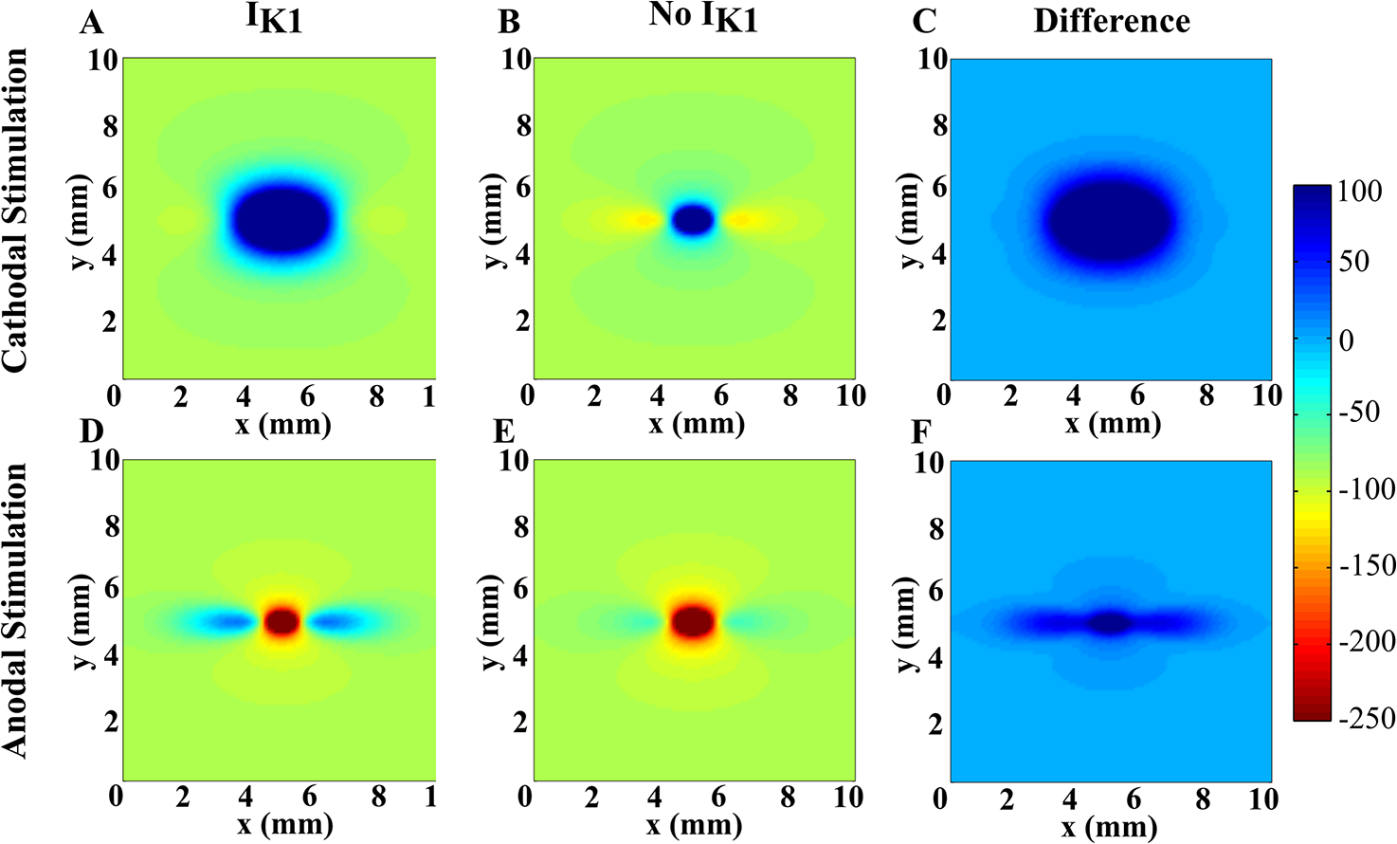
Steady-state *V_m_* as a function of position with and without *I_K1_*. The fibers are horizontal, the extracellular stimulating electrode is at the center of the tissue, and the time is 50 ms after application of the stimulus. Cathodal stimulation using *I_**0**_* = 4 mA/mm with (A) and without (B) *I_**K1**_*, and anodal stimulation using *I_**0**_* = - 4 mA/mm with (D) and without (E) *I_**K1**_*, are shown. The difference between *V_**m**_* with *I_**K1**_* and without *I_**K1**_* are shown in (C) and (F).

The steady-state transmembrane potential distribution without *I*_*K1*_ (Fig 2B and 2E) are similar to those presented by Sepulveda et al. [2]. The regions of depolarization and hyperpolarization are symmetrical with respect to the polarity of the stimulus. For cathodal stimulation, the tissue is strongly depolarized under the cathode and weakly hyperpolarized on each side of the cathode along the myocardial fibers (the *x* direction, horizontal). When we include *I*_*K1*_ (Fig 2A and 2D), the regions of depolarization grow and the regions of hyperpolarization shrink compared to the no *I*_*K1*_ case. The differences between the *I*_*K1*_ and the no *I*_*K1*_ cases are shown in Fig 2C and 2F. For cathodal stimulation the difference is large under the cathode where depolarization occurs, whereas for anodal stimulation the difference is pronounced in the virtual cathode regions on each side of the anode.

**Fig 3:**
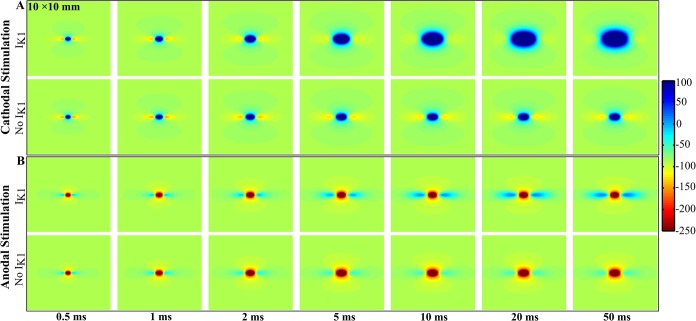
*V_m_* as a function of position in the bidomain model with and without *I_K1_* for different times. (A) Cathodal stimulus of *I_**0**_* = 4 mA/mm, and (B) anodal stimulus of *I_**0**_* = - 4 mA/mm. Movies of these simulations are included as S1 Movie, S2 Movie, S3 Movie, and S4 Movie.

Fig 3 shows the time evolution of the transmembrane potential. At short times (less than 2 ms) *V*_*m*_ is nearly the same whether or not the *I*_*K1*_ current is included in the model. For long times (greater than 2 ms) the steady-state behavior of *V*_*m*_ becomes prominent. Movies of this behavior are included in the supplementary file (cathode with *I*_*K1*_, S1 Movie; cathode with no *I*_*K1*_, S2 Movie; anode with *I*_*K1*_, S3 Movie; anode with no *I*_*K1*_, S4 Movie).

**Fig 4:**
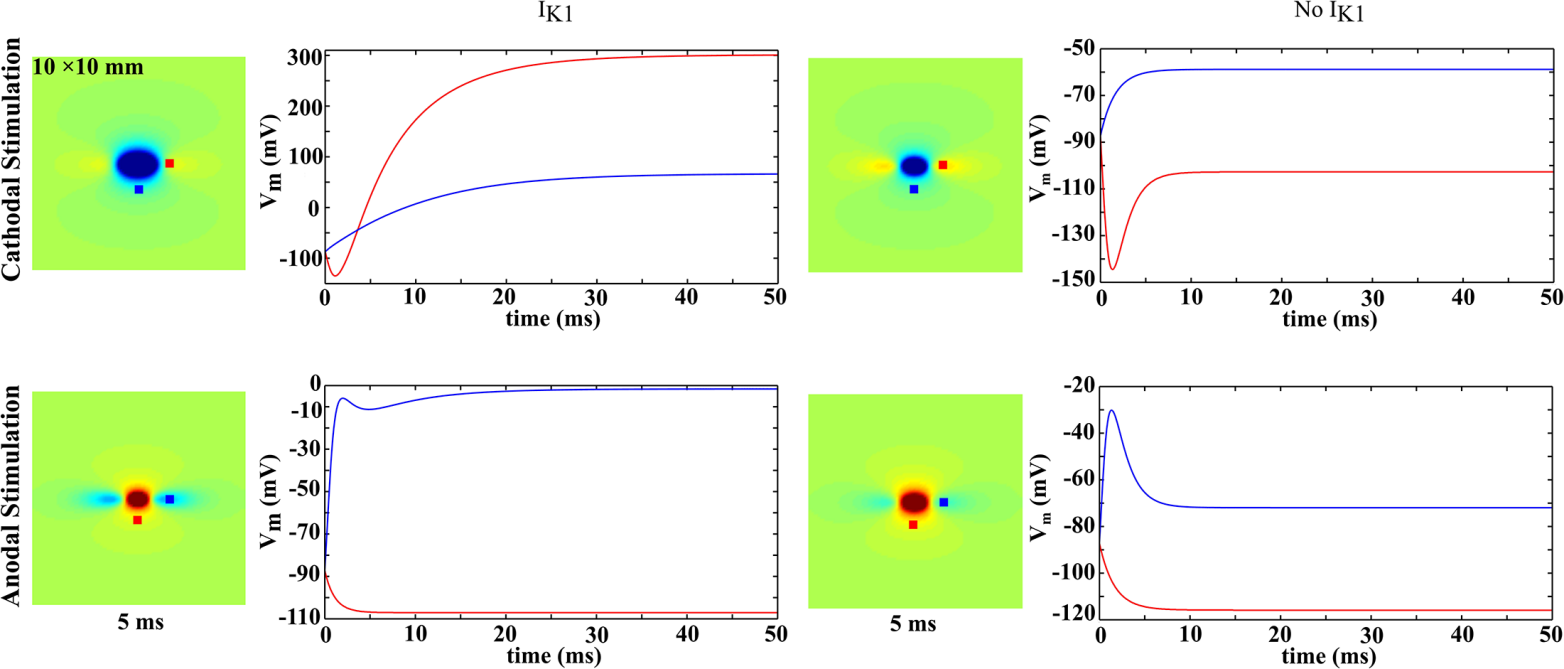
Time course of *V_m_* (mV) at two locations with *I_K1_* and without *I_K1_*. The color scale is the same as in Figs 2 and 3.

To further clarify the temporal behavior, Fig 4 shows the transmembrane potential as a function of time at two locations: one in a depolarized region and another in a hyperpolarized region. Without *I*_*K1*_, the curves for the two polarities are symmetrical for depolarization and hyperpolarization. The hyperpolarization at the virtual anode during cathodal stimulation is initially large, and then decays with time. With *I*_*K1*_, the initial hyperpolarization is present, but at longer times the depolarization diffuses outward, causing the point to be depolarized in steady state.

**Fig 5:**
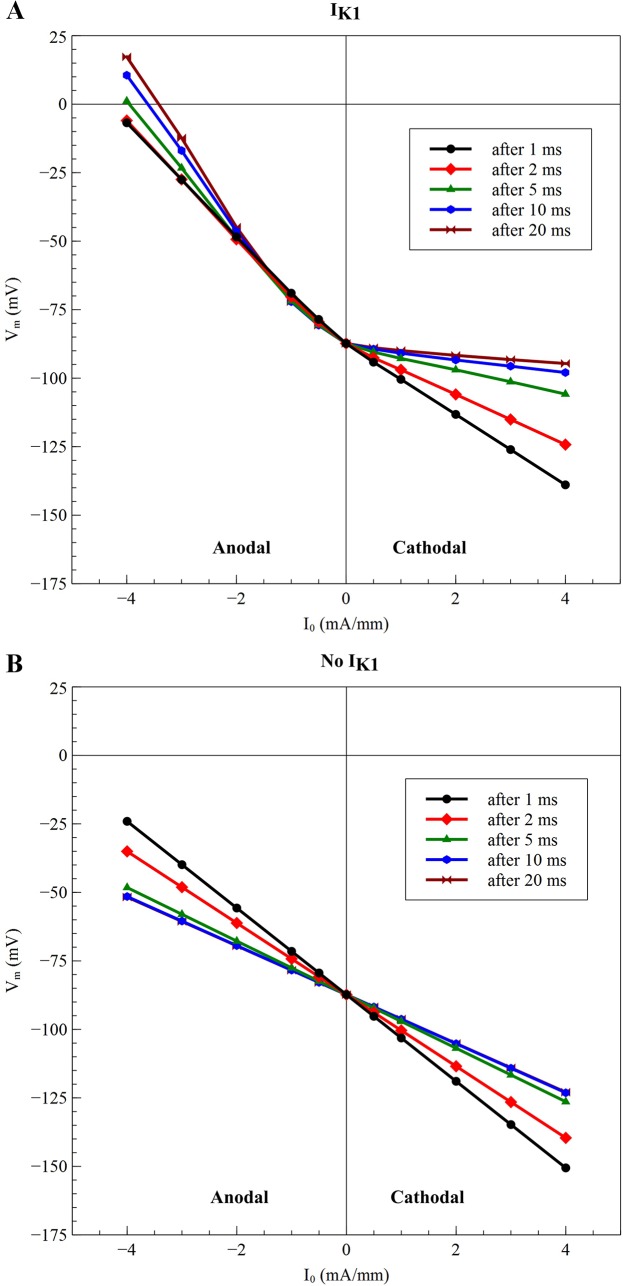
The peak value of hyperpolarization during a cathodal stimulus and the peak depolarization during an anodeal stimulus, with (A) and without (B) *I_K1_*. In (B), the 10 and 20 ms curves are nearly identical and difficult to distinguish. The times (1, 2, 5, 10, and 20 ms) were selected to highlight the behaviors on both slow and fast time scales. The stimulus currents (-4, -3, -2, -1, -0.5, 0, 0.5, 1, 2, 3, and 4 mA/mm) were selected to cover a range of weak and strong stimuli of both polarities.

For a bidomain without *I*_*K1*_ (Fig 5B) the peak hyperpolarization at the virtual anode for an anodal stimulus decreases with time, as does the depolarization at the virtual cathode for a cathodal stimulus. The current-voltage curves are linear, as expected from a linear model. Hyperpolarization at a virtual anode is just as prominent as depolarization at a virtual cathode

With *I_K1_* (Fig 5A) the virtual cathode depolarization during an anodal stimulus (negative values of *I_0_*) increases with time, and the virtual anode hyperpolarization during a cathodal stimulus (positive *I_0_*) decreases with time. This result is consistent with the calculations by Roth and Trayanova [16] and by Sambelashvili et al. [10]. The hyperpolarization at the virtual anode is suppressed dramatically for long duration pulses (20 ms), but the behavior is nearly linear for short durations (1 ms).

## Discussion

We have studied the effect of the *I*_*K1*_ inward-rectifying potassium current on an anisotropic two dimensional sheet of cardiac tissue using the bidomain model. Experiments reveal little or no hyperpolarization at a virtual anode when a shock is applied to resting tissue [6–8]. Another experiment showed that hyperpolarization appeared at the beginning of a shock but disappeared within 1 ms [17]. Our numerical simulations carried out using the bidomain model with *I*_*K1*_ indicate that the hyperpolarization in the virtual anode region is small compared to the depolarization at the corresponding virtual cathode. By comparing simulations with and without the *I*_*K1*_ potassium current, we conclude that *I*_*K1*_ significantly suppresses hyperpolarization at a virtual anode.

In our simulations using the *I_K1_* current, the hyperpolarization at the virtual anode developed very quickly and almost linearly for short pulse durations, but then decayed to a small value at long durations (Fig 5(A)). This behavior arises because initially the tissue responds to the stimulus by charging the membrane capacitance, and the model is linear if the membrane ionic current is neglected and only the capacitive current is included. Only at larger times, as the tissue approaches steady state, does the behavior of the ionic current become crucial, and then the impact of the inward rectification is apparent.

Our model includes only two currents: a linear current and *I_K1_*. Therefore, it cannot simulate action potential excitation and propagation, which would require at least the inclusion of a sodium current. We left out other currents because we wanted to focus exclusively on the impact of the inward rectification of the *I_K1_* current. Although our simulations cannot model excitation, they should be a good model for subthreshold stimuli, because *I_K1_* is the largest current when the transmembrane potential is near resting potential. Sambelashvili et al. [10] included the full Luo-Rudy model [11] with sodium, calcium, and other potassium currents, and found results similar to ours for subthreshold stimuli. If the stimulus is applied to refractory tissue (as discussed in [3]) then hyperpolarization at the virtual anode may be more apparent.

## Supporting Information

S1 Data All data used in this study. (ZIP)

S1 File FORTRAN computer code used to solve the bidomain equations. (TXT)

S1 Movie The transmembrane potential as a function of space and time, for cathodal stimulation with *i_K1_*. (MP4)

S2 Movie The transmembrane potential as a function of space and time, for cathodal stimulation without *i_K1_*. (MP4)

S3 Movie The transmembrane potential as a function of space and time, for anodal stimulation with *i_K1_*. (MP4)

S4 Movie The transmembrane potential as a function of space and time, for anodal stimulation without *i_K1_*. (MP4)
